# Characterisation of autophagy induction by the thiopurine drugs azathioprine, mercaptopurine and thioguanine in THP-1 macrophages

**DOI:** 10.1007/s00210-024-03563-0

**Published:** 2024-11-01

**Authors:** Connan D. Masson, Fern Findlay-Greene, Filipa Henderson Sousa, Paul Henderson, Jennifer A. Fraser, Peter G. Barlow, Craig Stevens

**Affiliations:** 1 School of Applied Sciences, Edinburgh Napier University, Sighthill Campus, Sighthill Court, Edinburgh, EH11 4BN UK; 2 Centre for Discovery Brain Sciences and UK Dementia Research Institute, The University of Edinburgh, Edinburgh, EH16 4SB UK; 3 Child Life and Health, University of Edinburgh, Edinburgh, EH16 4TJ UK; 4 Department of Paediatric Gastroenterology and Nutrition, Royal Hospital for Children and Young People, Edinburgh, EH16 4TJ UK; 5 Royal (Dick) School of Veterinary Studies, The University of Edinburgh, Easter Bush Campus, Edinburgh, EH25 9RG UK

**Keywords:** Thiopurines, Autophagy, MTORC1, EIF2α

## Abstract

Activating autophagy may be therapeutically beneficial, and we have previously shown that azathioprine (AZA), an immunomodulatory drug, induces autophagy. Here, we evaluated the induction of autophagy by the thiopurines AZA, mercaptopurine (6-MP) and thioguanine (6-TG) in THP-1 macrophages and investigated the mechanism of action in the context of this cellular process. The cytotoxicity of thiopurines was evaluated using an LDH assay. Induction of endogenous LC3 by thiopurines was evaluated using immunostaining. To confirm autophagy activation by thiopurines, a GFP-RFP-LC3 reporter plasmid was used to monitor the maturation of autophagosomes to autolysosomes. Induction of apoptosis by thiopurines was evaluated using Annexin V/PI staining, and ER stress was assessed via RT‒PCR analysis of *XBP1* splicing. To gain insight into the mechanism of action of thiopurines, mTORC1 activity and eIF2α-S51 phosphorylation were evaluated by immunoblotting. Thiopurines were not cytotoxic to cells and induced strong time- and concentration-dependent autophagy. Thiopurines activate autophagy with complete progression through the pathway. Induction of autophagy by thiopurines occurred independently of apoptosis and ER stress. Immunoblotting revealed that AZA inhibited mTORC1 activity, and AZA and 6-TG increased eIF2α-S51 phosphorylation. In contrast, 6-MP had a minor effect on either signalling pathway. Thiopurines are strong inducers of autophagy, and autophagy induction should be considered among the mechanisms responsible for patient response to thiopurines.

**Supplementary Information:**

The online version contains supplementary material available at 10.1007/s00210-024-03563-0.

## Introduction

The thiopurines azathioprine (AZA), mercaptopurine (6-MP), and thioguanine (6-TG) are immunomodulating drugs that are commonly used to treat cancer, inflammatory bowel disease (IBD), and other autoimmune diseases. Although thiopurine methyltransferase (*TPMT*) genotyping and thiopurine metabolite blood monitoring are now performed regularly in the clinic, up to one-third of patients have to discontinue thiopurines due to side effects (Warner et al. 2018), with 6-TG used less frequently than AZA or 6-MP due to its higher levels of cytotoxicity (Dubinsky 2004). It is well established that thiopurine metabolites can inhibit de novo purine biosynthesis, inhibit DNA/RNA synthesis, and deactivate proinflammatory T lymphocytes, thereby reducing inflammation by dampening the immune response (Karran and Attard 2008); however, their molecular mechanisms of action are not fully understood. Additional activities, including the modulation of autophagy (Chaabane and Appell 2016; Guijarro et al. 2012; Hernandez-Breijo et al. 2013; Hooper et al. 2019b; Zeng and Kinsella 2008, 2010; Zeng et al. 2007) and the inhibition of the small GTPase Rac1, (Marinkovic et al. 2014; Poppe et al. 2006; Tiede et al. 2003; Wildenberg et al. 2017), have been described, leading to the induction of T cell apoptosis, impairment of the interaction between T cells and antigen-presenting cells, and dampened proinflammatory function of macrophages.

Macrophages play an important role in the human immune system, carrying out various functions, including killing pathogens, and regulating lymphocyte activation and proliferation. Macrophage function plays a crucial role in the pathogenesis of many diseases, including IBD (Mahida 2000), and several genetic variants related to autophagy have been identified as susceptibility factors that could directly influence macrophage function (Buisson et al. 2019; Lapaquette et al. 2012).

Autophagy is an evolutionarily conserved intracellular degradation pathway that recycles misfolded proteins and aged or damaged organelles via the lysosome (Klionsky et al. 2021b). It functions at a basal level to maintain cellular homeostasis and is induced in response to myriad stresses, including starvation, DNA damage, and infection (Klionsky et al. 2021b). Autophagy plays an important role in promoting cell survival in response to stress, and emerging evidence supports the use of autophagy modulators in disease treatment (Aman et al. 2021; Kocak et al. 2022). In this study, we aimed to characterise the effects of thiopurines on autophagy in THP-1 macrophages.

## Materials and methods

### Cell culture and transfection

THP-1 monocytes were grown in RPMI 1640 media (Sigma-Aldrich, Irvine, UK) supplemented with 10% heat-inactivated foetal bovine serum (FBS) (Gibco, Thermo Fisher Scientific, Paisley, UK), 1% L-glutamine (Gibco), and 1% penicillin–streptomycin (Gibco, product number 15140122). For differentiation into macrophages, THP-1 monocytes were incubated in RPMI supplemented with 20 ng/mL phorbol myristate acetate (PMA) (Tocris, Bristol, UK) for 48 h and then rested for 24 h in fresh RPMI before the experiments.

For transfection of THP-1 macrophages, 5 × 10^5^ cells were seeded in 6-well plates. Adherent cells were detached from tissue culture plates using trypsin (Gibco), collected in 15-mL Falcon tubes (Falcon, Corning International, USA), and centrifuged at 250*xg* at room temperature for 10 min (Pendragon, Scientific Ltd., Buckingham, UK). The supernatant was aspirated, and the cell pellets were resuspended in 100 µL of nucleofector solution (Amaxa Cell Line Nucleofector Kit V) according to the manufacturer’s instructions (Lonza, Manchester, UK). The GFP-RFP-LC3 plasmid (0.5 µg) was added to the cell suspension, gently mixed, and transferred to a nucleofector cuvette (Lonza). The cells were electroporated using the Y-001 programme according to the manufacturer’s instructions with the Nucelofector 2b device (Lonza). Fresh RPMI (500 µL) was added to the transfected cells, and the cell suspension was combined with an additional 1 mL of RPMI in a 6-well plate. The cells were incubated for 24 h before analysis or further treatment.

### Cell treatments

The pharmacological agents were resuspended in dimethyl sulfoxide (DMSO; Sigma-Aldrich) and diluted to a working concentration in cell culture media. The final concentration of DMSO in the media was ≤ 0.2%. Details of the pharmacological agents used in this study are provided in Table 1. For serum starvation, cells were incubated with RPMI 1640 medium that did not contain FBS.

**Table 1:** Pharmacological agent

Agent	Stock conc	Working conc	Supplier/Cat. No
Azathioprine	100 mM	20–120 µM	Tocris/4099
Mercaptopurine	100 mM	20–120 µM	Tocris/4103
Thioguanine	100 mM	20–120 µM	Tocris/4061
Phorbol 12-myristate 13-acetate	1 mg/mL	25 nM	Tocris/1201
Brefeldin A	5 mg/mL	10 µM	Tocris/1231
Bafilomycin A1	1 mM	160 nM	Cell signalling/54645
Rapamycin	100 µM	100 nM	Cell signalling/9904

### LDH assay

THP-1 macrophages were seeded at 2 × 10^4^ in 96 well plates and treated with media supplemented with thiopurines for 2 h or 8 h. At the end of the incubation, 50 µL of supernatant from each well was transferred to a new 96-well plate and incubated with 50 µL of CytoTox 96 reagent (Promega, Madison, WI, USA) for 30 min at RT. Stop solution (50 µL) was added to each well, and the absorbance was measured at 490 nm using a plate reader (Tecan Sunrise, Männedorf, Switzerland). Controls were generated according to the manufacturer’s recommendations.

### Immunostaining

THP-1 macrophages were seeded at 5 × 10^5^ on 22 mm borosilicate glass coverslips (VWR International) or 35 mm imaging dishes (Ibidi, Thistle Scientific, Uddingston, UK) for 24 h before treatment. For immunostaining, cells were fixed with 4% paraformaldehyde (PFA) in PBS for 15 min, permeabilised with PBS/0.1% Triton X-100 (Sigma-Aldrich), and blocked with PBS containing 10% goat serum (Gibco). Primary antibodies were incubated overnight at 4 °C in a humidified chamber, and the sections were incubated with conjugated secondary antibodies for 1 h at RT. The details of the antibodies used in this study are provided in Supplementary Table 1. Where appropriate, the cells were counterstained with Vectashield mounting medium containing 4′,6′-diamidino-2-phenylindole (DAPI) (Vector Laboratories, Peterborough, UK). Images were captured using a Carl Zeiss LSM880 confocal microscope (Jena, Germany) and analysed using ImageJ software (National Institutes of Health, Bethesda, MD, USA).

### Autophagy assays

#### Endogenous LC3 assay

To evaluate the induction of autophagy, THP-1 macrophages were immunostained for the specific marker protein LC3-II (Klionsky et al. 2021a). The basal threshold number of LC3-II puncta was established by manually counting the puncta in 30 cells from 3 fields of view and calculating the average puncta count per cell. Enhanced autophagy activity was established in each condition by manually counting the puncta in thirty cells from three fields of view and determining the percentage of cells exhibiting puncta above the basal threshold number.

#### Tandem fluorescent-tagged GFP-RFP-LC3 assay

THP-1 macrophages were transiently transfected with the GFP-RFP-LC3 plasmid (Klionsky et al. 2021a). This plasmid utilises the difference in pH between the acidic autolysosome (formed by the fusion of an autophagosome and lysosome) and the neutral autophagosome, with differences in pH sensitivity exhibited by GFP (labile at acidic pH) and RFP (stable at acidic pH). Thus, this plasmid can be used to monitor the maturation of autophagosomes (RFP + GFP +) to autolysosomes (RFP + GFP-). The number of RFP + GFP + puncta versus RFP + GFP- puncta per cell was established in each condition by manually counting 10 cells and calculating the average puncta count per cell.

### Annexin/PI staining

THP-1 macrophages were seeded at 5 × 10^5^ in 6-well plates and stained using the FITC Annexin V/PI Apoptosis Detection Kit (BioLegend, London, UK). The cells were detached from the dish using trypsin for 3 min and transferred to a flow cytometry tube. The cells were washed twice in PBS, counted, and resuspended in the supplied Annexin-V binding buffer at a concentration of 1 × 10^6^/mL. The cell suspension (100 μL) was transferred to a 5-mL flow cytometry tube, after which 5 μL of FITC-Annexin-V and 10 μL of propidium iodide (PI) solution were added to the cell suspension. The cells were gently vortexed and incubated for 15 min at RT in the dark. Annexin-V binding buffer (400 μL) was added, and the samples were analysed on a flow cytometer (FACSCelesta, BD Biosciences). Experimental staining controls were used to set the gating parameters and voltage compensation. The threshold for all treatment groups was set at 5000 events, which were stopped after 10,000 events. The staining controls included unstained cells, cells stained with Annexin V only, and cells stained with PI only to determine compensation and set the gates on the cell populations. Annexin-V fluorescence was measured at an excitation wavelength of 495 nm and an emission wavelength of 525 nm (FITC). PI fluorescence was measured at an excitation wavelength of 488 nm and an emission wavelength of 610 nm (Texas Red). Cell viability was assessed through the fluorescence of Annexin and PI, with Annexin V-/PI- cells grouped as viable cells, Annexin V + /PI- cells grouped as early apoptotic cells, Annexin V + /PI + cells grouped as late apoptotic cells, and Annexin V-/PI + cells grouped as necrotic cells.

### RT-PCR analysis of XBP1 splicing

THP-1 macrophages were seeded at 1 × 10^6^ in 60-mm dishes. RNA was extracted from treated THP-1 macrophages using Trisure Reagent (Thermo Fisher Scientific) according to the manufacturer’s instructions. RNA was quantified using a Nanodrop 2000 (Thermo Fisher Scientific), and 2 µg of RNA was reverse transcribed using an RNA-to-cDNA high-capacity kit (AB systems, Winghall, UK). The control cDNA reactions lacked the RT enzyme (-RT).

PCR (10 µL) for evaluating *actin* and *XBP1* expression contained 25 ng and 50 ng of cDNA templates, respectively; 1X MangoMix™ (Meridian Bioscience, Memphis, TN, USA); and 100 pm/µl of forward and reverse oligonucleotides (Actin FP: GGGAAATCGTGCGTGACATT; RP: CCACAGGACTCCATGCCC; XBP FP: 5′-GGAGTTAAGACAGCGCTTGGGGA-3′; XBP RP: 5′-TGTTCTGGAGGGGTGACAACTGGG-3′). Control PCRs contained templates from -RT reactions to evaluate genomic DNA contamination or DEPC-treated deionised H_2_O as a substitute for the cDNA template (no template control, NTC) to evaluate contamination of reagents. Thirty-five cycles of denaturation (94 °C for 15 s), annealing (56 °C for 15 s), and amplification (72 °C for 20 s) were performed in a 2720 thermocycler (Applied Biosystems). Amplicons were resolved by 1% agarose gel electrophoresis containing 1% SafeView NBS Biologicals (Cambridgeshire, UK) and visualised using a G:Box System (Syngene, Cambridge, UK).

### Immunoblotting

THP-1 macrophages were seeded at 1 × 10^6^ in 60-mm dishes. After treatment cells were harvested by scraping into ice-cold phosphate-buffered saline (PBS) (Sigma‒Aldrich), transferring them into Eppendorf tubes, and centrifuging them at 14,000*xg* for 15 min at 4 °C. The supernatant was removed, and the cell pellets were lysed in RIPA buffer (Thermo Fisher Scientific) containing 1 × Halt Protease Inhibitor Cocktail (Thermo Fisher Scientific, product number 87786) and 1 × Phosphatase Inhibitor Cocktail (Cell Signalling Technology) for 30 min, followed by centrifugation at 14,000*xg* for 15 min at 4 °C. The protein concentration of the soluble lysate was measured using a BCA assay (Thermo Fisher Scientific) following the manufacturer’s instructions, and the absorbance was measured at 490 nm using a plate reader. Protein lysates (20 μg) were resolved by reducing and denaturing electrophoresis on SDS-PAGE gels and electro-transferred to 0.45 μm nitrocellulose membranes (GE HealthCare Life Science, Amersham, UK). The membranes were blocked in PBS-5% BSA for 30 min and then incubated with primary antibody overnight at 4 °C in PBS-5% BSA. After washing (3 × 10 min) in PBS-0.1% Tween-20, the membranes were incubated with a secondary antibody for 1 h at room temperature (RT) in PBS-5% BSA. Proteins were analysed using an Odyssey imaging system and Image Studio 2.0 Software (LI-COR Biosciences, Nebraska, USA).

### Statistical analysis

Data are presented as mean ± SEM of three independent experiments. Statistical analysis was performed using one-way or two-way ANOVA with Dunnett’s post-test with GraphPad Prism 8 (GraphPad Software, La Jolla, CA, USA). A *p v*alue ≤ 0.05 was considered statistically significant.

## Results

### Cytotoxicity of thiopurines

**Fig. 1:**
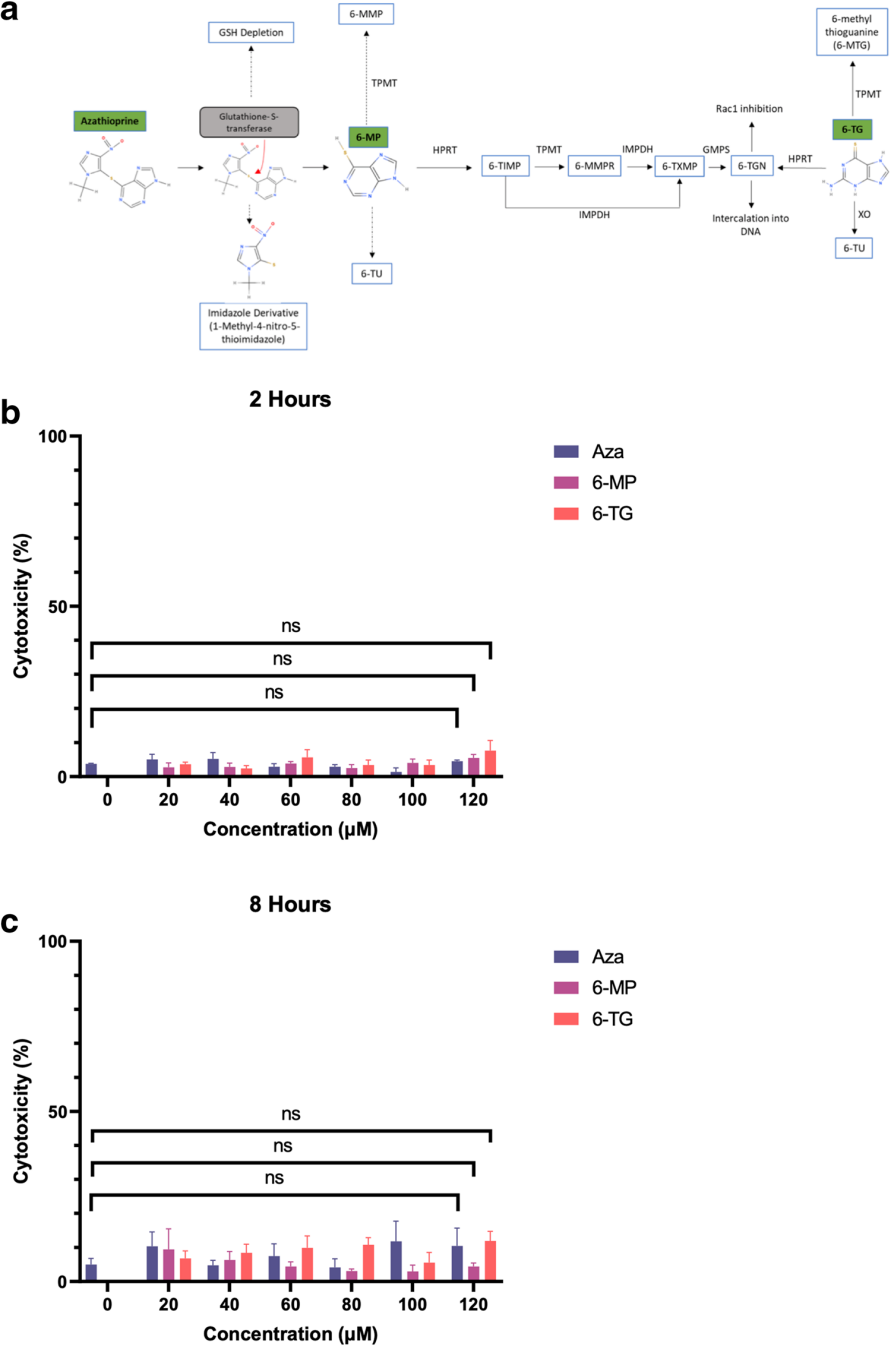
Cytotoxicity of thiopurines. **a** Schematic illustrating the metabolism of thiopurines to 6-thioguanine nucleotides (6-TGNs). Abbreviations: 6-methyl mercaptopurine (6-MMP), 6-methyl mercaptopurine ribonucleotide (6-MMPR), 6-thioxanthosine monophosphate (6-TXMP), hypoxanthine–guanine phosphoribosyl transferase (HPRT), inosine-5′-monophosphate dehydrogenase (IMPDH), glutathione (GSH), glutathione-S-transferase (GST), guanosine monophosphate synthetase (GMPS), thioinosine monophosphate (6-TIMP), thiopurine methyltransferase (TPMT), thiouric acid (TU), xanthine oxidase (XO). **b** THP-1 macrophages were treated with different concentrations of thiopurines ranging from 20 to 120 μM, and cytotoxicity was measured using the LDH assay at 2 h (*n* = 3). ns = not significant (*p* > 0.05). **c** THP-1 macrophages were treated with different concentrations of thiopurines ranging from 20 to 120 μM, and cytotoxicity was measured using the LDH assay at 8 h (*n* = 3). ns = not significant (*p* > 0.05)

AZA is converted to 6-MP through conjugation with glutathione (GSH) in a reaction catalysed by glutathione-S-transferase (GST). 6-MP then undergoes a series of enzyme-catalysed reactions to form the active metabolite thioguanine nucleotides (6-TGNs). In contrast, 6-TG is directly metabolised to 6-TGN by hypoxanthine–guanine phosphoribosyl transferase (HPRT) (Fig. 1A). Thiopurines are associated with cytotoxicity (Fotoohi et al. 2010), and the induction of autophagy is intrinsically linked with physiological stress (Klionsky et al. 2021b). We aimed to determine whether thiopurines have a direct effect on autophagy induction; therefore, it was important to conduct our experiments under conditions where physiological stress responses were not activated to ensure they did not influence autophagy induction. In a previous study (Hooper et al. 2019b), we evaluated the modulation of autophagy by IBD drugs and demonstrated autophagy induction with AZA over a 12 h time course and corresponding concentration range (20–120 μM). Importantly, AZA was not cytotoxic to cells under these conditions. Therefore, we reasoned a similar time course and concentration range would apply to thiopurines in a THP-1 cell model. In the present study, the cytotoxicity of thiopurines was measured in THP-1 macrophages using the LDH assay. At 2 h (Fig. 1B) and 8 h (Fig. 1C), thiopurines were not cytotoxic to cells across the concentration range, and there was no significant change (*p* > 0.05) at the highest concentration tested (120 μM).

### Thiopurines induce autophagy

**Fig. 2:**
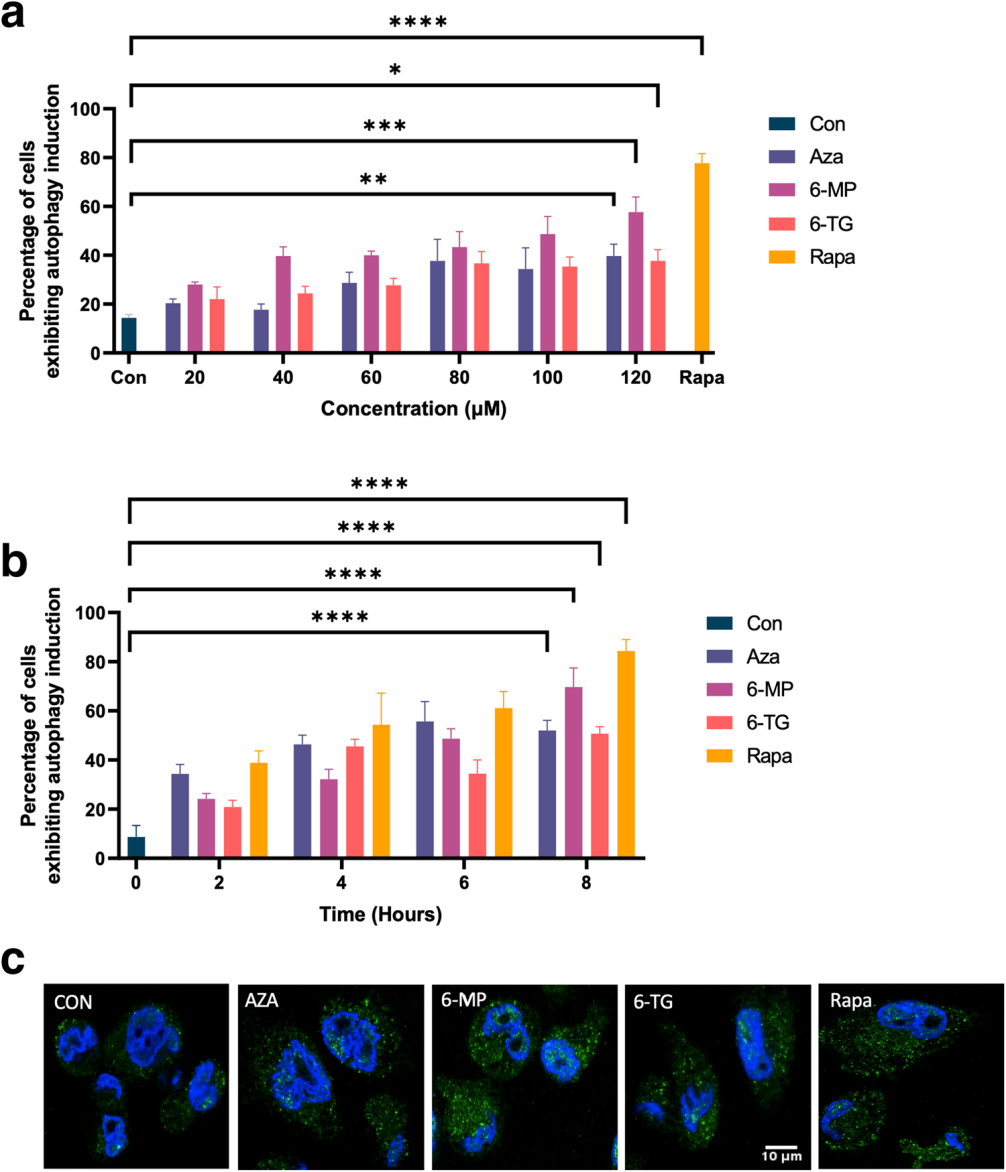
Thiopurines induce autophagosome accumulation. **a** THP-1 macrophages were treated with different concentrations of thiopurines ranging from 20 to 120 μM or 100 nM rapamycin (Rapa) for 8 h, and the accumulation of autophagic puncta was assessed by immunostaining for LC3-II (*n* = 3) (*p ≤ 0.05, **p ≤ 0.01, ***p ≤ 0.001, ****p ≤ 0.0001). **b** THP-1 macrophages were treated with a fixed concentration of thiopurines (120 μM) or 100 nM rapamycin (Rapa) for different durations, and the accumulation of autophagic puncta was assessed by immunostaining for LC3-II (*n* = 3) (****p ≤ 0.0001). **c** THP-1 macrophages were treated with 120 μM thiopurine or 100 nM rapamycin (Rapa) for 8 h, and the accumulation of autophagic puncta was assessed by immunostaining for LC3-II. Representative images are shown (*n* = 3)

To determine the optimum conditions for autophagy induction, THP-1 macrophages were treated for 8 h with different concentrations of thiopurines or with a fixed concentration of thiopurines (120 μM) for various durations, and autophagosome accumulation was assessed by immunostaining for the autophagy-specific marker protein LC3-II. At the lowest concentration tested (20 μM), 20% of the cells exhibited an accumulation of autophagosomes in response to AZA, 28% in response to 6-MP, and 22% in response to 6-TG. At the highest concentration tested (120 μM), 40% of the cells exhibited significant autophagy induction in response to AZA (*p* ≤ 0.01), 58% in response to 6-MP (*p* ≤ 0.001), and 38% in response to 6-TG (*p* ≤  0.05) (Fig. 2A). At the shortest time point tested (2 h), 34% of the cells exhibited an accumulation of autophagosomes in response to AZA, 24% in response to 6-MP, and 21% in response to 6-TG. At the longest time point tested (8 h), 52% of the cells exhibited significant autophagy induction in response to AZA (*p* ≤  0.0001), 70% in response to 6-MP (*p* ≤  0.0001), and 51% in response to 6-TG (*p* ≤ 0.0001) (Fig. 2B). Rapamycin, an acute inhibitor of mTOR complex 1 (mTORC1) (Lamming 2016), significantly (*p* ≤  0.0001) induced autophagy. These results demonstrated strong time- and concentration-dependent induction of autophagy in response to thiopurines. Based on these results, the 120 μM concentration and 8 h time point were selected for subsequent experiments; representative immunostaining images obtained using these treatment conditions are presented in Fig. 2C.

### Thiopurines activate autophagy

**Fig. 3:**
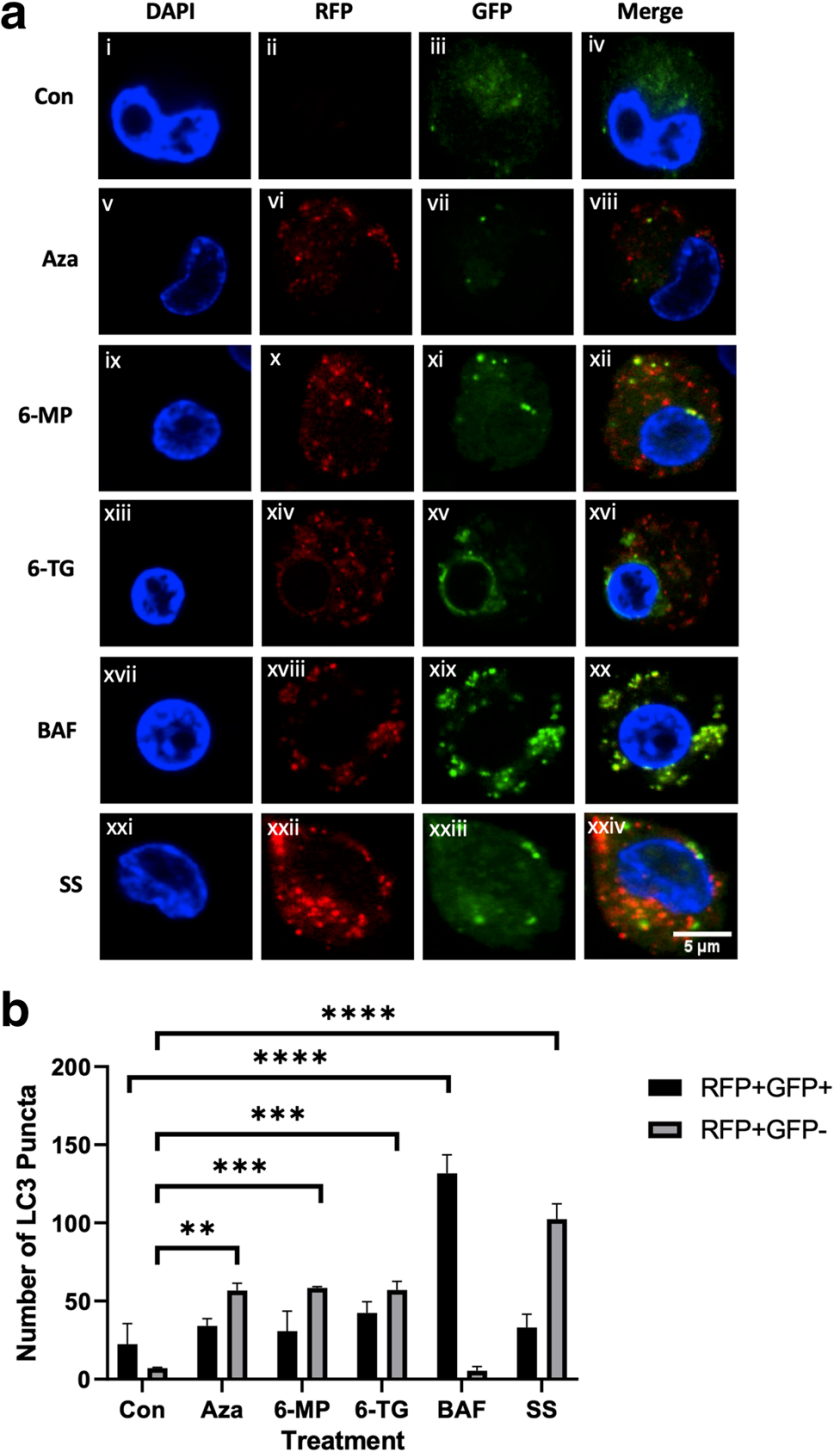
Thiopurines activate the autophagy pathway. **a** THP-1 macrophages were transfected with a GFP-RFP-LC3 plasmid and treated with 120 μM thiopurine, 160 nM bafilomycin A1 (BAF), or serum starved (SS) for 8 h. Representative confocal images are shown (*n* = 3). **b** Quantification of cells exhibiting neutral autophagosomes (RFP + GFP +) or acidified autolysosomes (RFP + GFP-) in thiopurine-treated, bafilomycin A1 (BAF)-treated, or serum-starved (SS) cells. The number of RFP + GFP + puncta versus RFP + GFP- puncta per cell in each condition was counted (3 cells) (*n* = 3) (**p ≤ 0.01, ***p ≤ 0.001, ****p ≤ 0.0001)

Autophagosomes and LC3-II can accumulate due to activation or inhibition of the autophagy pathway. We transiently transfected THP-1 macrophages with the GFP-RFP-LC3 plasmid to monitor progression through the pathway (Klionsky et al. 2021a). Treatment of transfected cells resulted in significant accumulation of RFP + GFP- puncta with AZA (*p* ≤  0.01) Fig. 3A,vi), 6-MP (*p* ≤  0.001) Fig. 3A,x), and 6-TG (*p* ≤  0.001) Fig. 3A,xiv) and quantified in Fig. 3B, indicating that complete progression through the pathway had occurred. A significant (*p* ≤  0.0001) increase in RFP + GFP- puncta was also observed with serum starvation (Fig. 3A,xxii). Inhibition of autophagosome-lysosome fusion with bafilomycin A1 (Klionsky et al. 2021a) resulted in a significant (*p* ≤  0.0001) accumulation of RFP + GFP + puncta, with almost complete colocalisation observed, as indicated in the merged image (Fig. 3A,xx).

### Effect of thiopurines on apoptosis

**Fig. 4:**
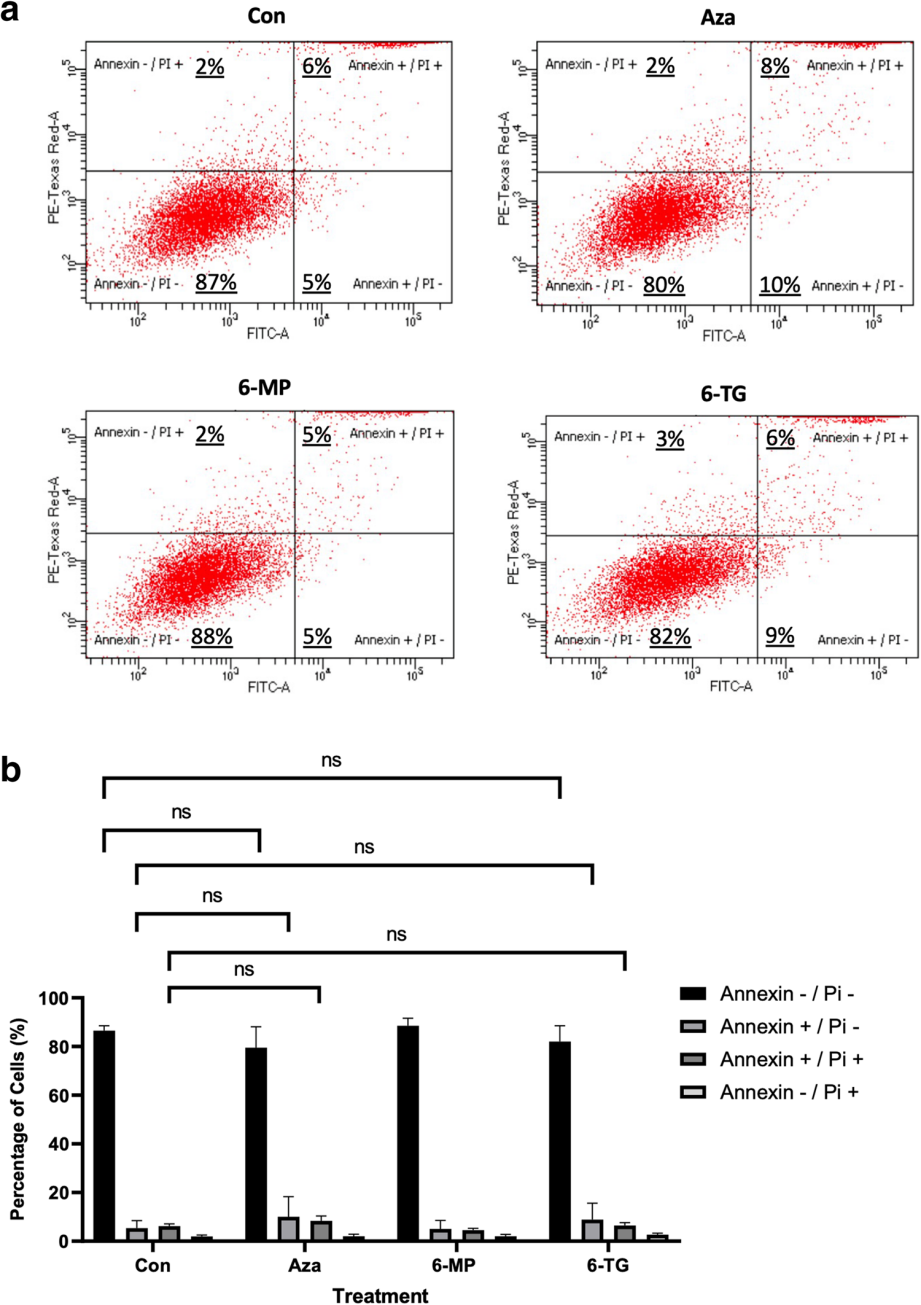
Thiopurines do not induce apoptosis. **a** THP-1 macrophages were treated with 120 µM thiopurines for 8 h, stained using an Annexin-V kit and PI solution and analysed via flow cytometry. Representative dot plots are shown. The percentages in each quadrant are reported as the mean  of three independent experiments. **b** Quantification of the percentage of viable (Annexin-/PI-), early apoptotic (Annexin + /PI), late apoptotic (Annexin + /PI +), and necrotic (Annexin-/PI +) cells among control and thiopurine-treated cells (*n* = 3). ns = not significant (*p* > 0.05)

Due to the close relationship between autophagy and apoptosis, we investigated the effect of thiopurines on the early and late stages of apoptosis using Annexin-V/PI staining (Fig. 4A and B). When THP-1 macrophages were treated with thiopurines, there was no significant change (*p* > 0.05) in the percentage of healthy cells (Annexin-/PI-) **(**control (87%), AZA (80%), 6-MP (88%), 6-TG (82%)), the percentage of cells in early apoptosis (Annexin + /PI-) (control (5%), AZA (10%), 6-MP (5%), 6-TG (9%)), or the percentage of cells in late-stage apoptosis (Annexin + /PI +) (control (6%), AZA (8%), 6-MP (5%), 6-TG (6%)).

### Effect of thiopurines on ER stress

**Fig. 5:**
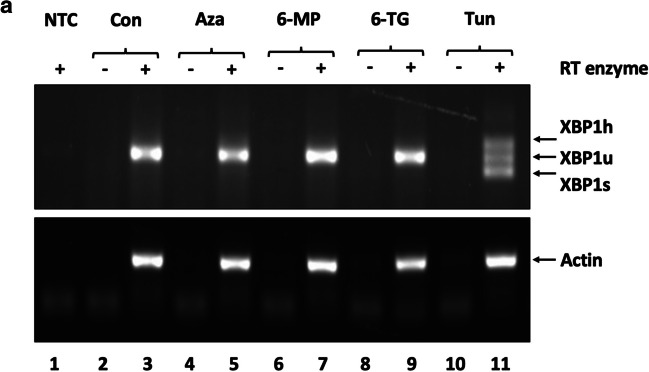
Thiopurines do not induce ER stress. **a** THP-1 macrophages were treated with 120 µM thiopurine or 5 µg/mL tunicamycin (Tun) for 8 h. The extracted RNA was reverse transcribed into cDNA before *XBP1*, and *actin* was amplified by RT‒PCR. The control PCRs included templates from reactions lacking the RT enzyme (-RT) or no template (NTC). PCR amplicons corresponding to XBP1h, XBP1u, and XBP1s are indicated. Representative images are shown (*n* = 3)

Autophagy and ER stress are intrinsically linked (Hooper et al. 2019a); therefore, we assessed the effect of thiopurines on ER stress in THP-1 macrophages using RT-PCR for the splicing of *XBP1*, a well-characterised marker of ER stress (Yoshida et al. 2001). *XBP1* splicing was not observed in response to AZA, 6-MP, or 6-TG treatment (Fig. 5A(5, 7 and 9), respectively). In contrast, tunicamycin, an inhibitor of *N-*linked glycosylation that results in the accumulation of misfolded proteins in the ER (Kishino et al. 2017), caused clear splicing of *XBP1* (Fig. 5A(11)). Two variants of *XBP1* were observed in response to tunicamycin treatment, which results from the formation of a hybrid double-stranded cDNA product (XBP1h) that consists of a single strand of unspliced *XBP1* (XBP1u) and a single strand of spliced *XBP1* (XBP1s) (Chalmers et al. 2017).

### AZA inhibits mTORC1 activity

**Fig. 6:**
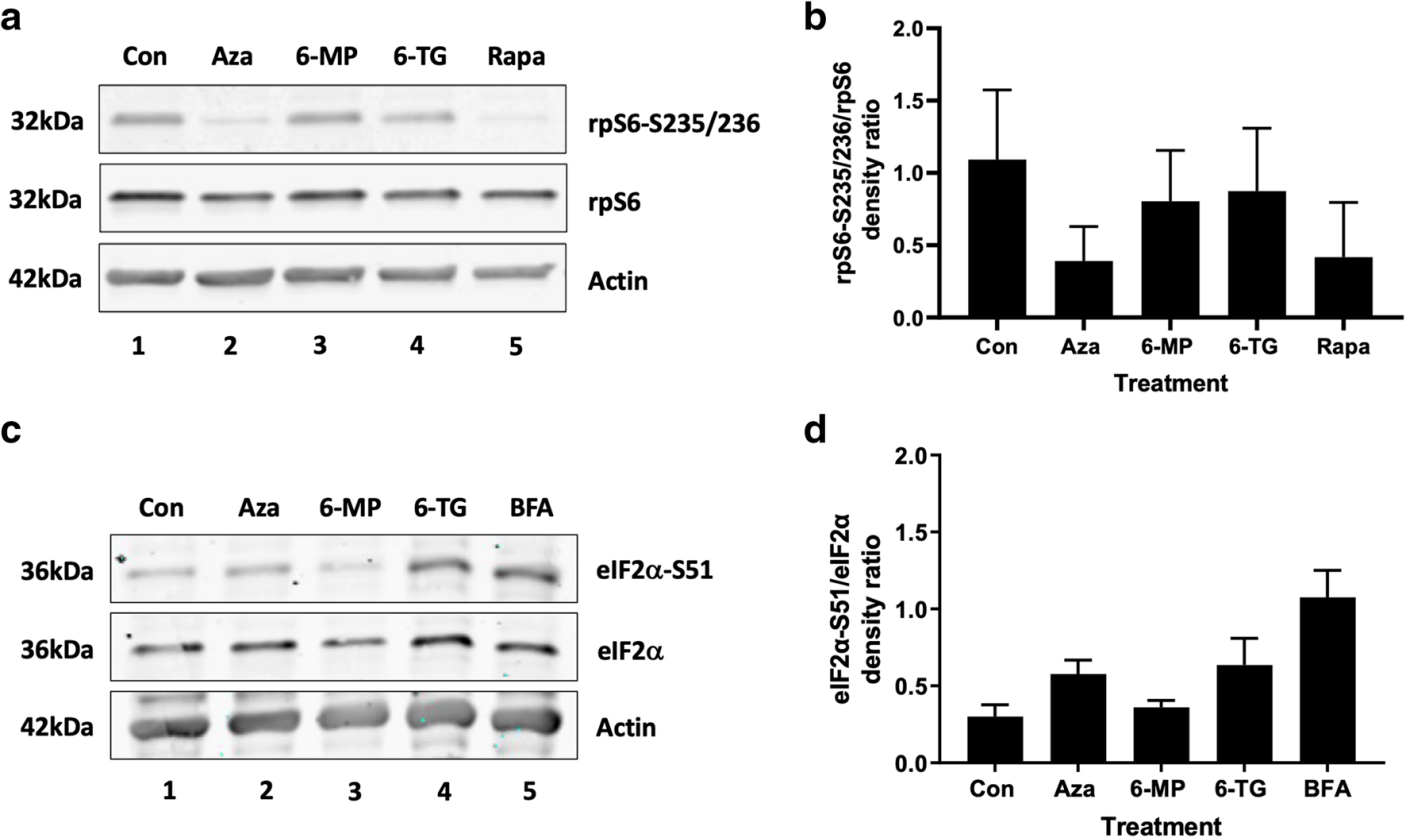
Thiopurines regulate the mTORC1 and eIF2α signalling pathways. **a** THP-1 macrophages were treated with 120 µM thiopurine or 100 nM rapamycin (Rapa) for 8 h. Protein samples were prepared and immunoblotted using antibodies specific for rpS6-S235/236, total rpS6, and total actin. Representative immunoblots are shown (*n* = 3). **b** Quantification of rpS6-S235/236 phosphorylation. Densitometry was performed on immunoblots using ImageJ software. The rpS6-S235/236 intensity was normalised to the total rpS6 intensity. The results are reported as the mean ± SEM of three independent experiments. **c** THP-1 macrophages were treated with 120 µM thiopurine or 10 µM brefeldin A (BFA) for 8 h. Protein samples were prepared and immunoblotted using antibodies specific for eIF2α-S51, total eIF2α, and actin. Representative immunoblots are shown (*n* = 3). **d** Quantification of eIF2α-S51 phosphorylation. Densitometry was performed on immunoblots using ImageJ software. The eIF2α-S51 intensity was normalised to the total eIF2α intensity. The results are reported as the mean ± SEM of three independent experiments

mTORC1 is a master regulator of cellular metabolism and autophagy (Liu and Sabatini 2020). The phosphorylation of ribosomal protein S6 at serine 235/236 (rpS6-S235/236) is a well-characterised surrogate marker of mTORC1 activity (Laplante and Sabatini 2012). Immunoblotting of THP-1 macrophage lysates revealed a notable decrease in rpS6-S235/236 phosphorylation following AZA treatment (Fig. 6A and B, Supplementary Fig. 1), with only minor changes observed with 6-TG and 6-MP. Rapamycin treatment decreased phosphorylated rpS6-S235/236.

### 6-TG and AZA increase the phosphorylation of eIF2α at serine 51

In response to diverse stimuli, eukaryotic cells activate a common adaptive pathway called the integrated stress response (ISR) (Pakos-Zebrucka et al. 2016). Eukaryotic initiation factor-2α (eIF2α) phosphorylation at serine 51 (eIF2α-S51) is central to the ISR. Immunoblotting of THP-1 macrophage lysates revealed a notable increase in eIF2α-S51 phosphorylation following AZA and 6-TG treatment (Fig. 6C and D, Supplementary Fig. 2), with only a minor change observed with 6-MP. Brefeldin A (BFA), an inhibitor of intracellular vesicle formation and protein trafficking between the ER and Golgi apparatus (Colanzi et al. 2013), increased phosphorylated eIF2α-S51.

## Discussion

Defective autophagy has been strongly linked to numerous diseases (Klionsky et al. 2021b), and evidence suggests that activating autophagy may be therapeutically beneficial (Massey et al. 2008); however, little is known about how thiopurine drugs currently approved for clinical use modulate autophagy. Making the best use of drugs already approved for clinical use is essential; therefore, we aimed to evaluate the effect of thiopurines on the autophagy pathway in THP-1 macrophages.

We demonstrated strong time- and concentration-dependent induction of autophagy in response to thiopurines. Autophagosomes and LC3-II can accumulate due to activation or inhibition of the autophagy pathway (Klionsky et al. 2021a); therefore, we utilised a GFP-RFP-LC3 reporter plasmid and observed an increase in RFP + /GFP- autophagic puncta in response to AZA, 6-MP, and 6-TG, confirming that activation and complete progression through the pathway occurred.

Autophagy induction by thiopurines has been previously demonstrated in various cell types, including HePG2, HCT116, HT29, human endometrial cancer (HEC59), human embryonic kidney (HEK293), THP-1, and peripheral blood mononuclear cells (PBMC) (Chaabane and Appell 2016; Guijarro et al. 2012; Hernandez-Breijo et al. 2013; Hooper et al. 2019b; Zeng & Kinsella 2008, 2010; Zeng et al. 2007). In these studies, cells were treated with thiopurine concentrations ranging from 3 to  120 μM for periods between 6 h and 5 days. In the present study, we exposed THP-1 cells to 120 μM of thiopurines for 8 h. We then assessed cytotoxicity, apoptosis and ER stress to ensure that they did not influence autophagy induction, as autophagy is linked to these cellular responses. Importantly, under these conditions, thiopurines were not cytotoxic to cells and did not induce apoptosis or ER stress. These results suggest that thiopurine metabolites did not accumulate to toxic concentrations in our cells. While the in vivo metabolism of thiopurines is well characterised, there is very little information available in cell lines. The time required for thiopurines to be metabolised is an important consideration, and future studies of metabolite analysis must be undertaken to determine the concentration in cell lines and whether this is comparable to concentrations found in the blood of patients undergoing thiopurine treatment. There is also a lack of data on thiopurine activities in their native state.

In several studies, thiopurine-induced autophagy coincided with the induction of apoptosis (Chaabane and Appell 2016; Zeng and Kinsella 2008, 2010; Zeng et al. 2007), and it has been suggested that autophagy induction is a survival mechanism to counter thiopurine cytotoxicity. It was shown that 6-TG induced apoptosis and autophagy via activation of Tumour protein P53 (p53) (Zeng et al. 2007) and mTORC1- S6K1 signalling pathways (Zeng and Kinsella 2008). The latter contrasts the established role of mTORC1 in autophagy inhibition, and the authors suggest that activation of mTORC1 may increase the translation of proteins required for autophagy induction. The same research group also demonstrated that Bcl2 and adenovirus E1B Nineteen-kilodalton Interacting Protein (BNIP3) mediates 6-TG-induced autophagy in a p53- and mTORC1-dependent manner (Zeng and Kinsella 2010). In the study by Chaabane and Appell, the authors suggest that induction of autophagy by thiopurines antagonises apoptosis by degrading damaged mitochondria through mitophagy (Chaabane and Appell 2016). Hernandez-Breijo and colleagues showed AZA-induced autophagy via a mechanism involving mTORC1-S6K1 and IGF-1 signalling; AZA-induced autophagy led to cell cycle arrest; however, the cells died by apoptosis when AZA was combined with the autophagy inhibitor bafilomycin A1 (Hernandez-Breijo et al. 2013). In a previous study (Hooper et al. 2019b), we showed that AZA induces autophagy independent of apoptosis via mechanisms involving modulation of mTORC1 signalling and stimulation of the unfolded protein response (UPR) sensor protein kinase RNA-like ER kinase (PERK). It is clear from these studies that thiopurines activate complex and convergent signalling pathways to regulate autophagy.

Consistent with our previous study (Hooper et al. 2019b), we showed that AZA inhibits mTORC1 activity. mTORC1 activation enhances the synthesis of proteins and other macromolecules required for cell growth while inhibiting catabolic processes, such as autophagy and lysosome biogenesis (Liu and Sabatini 2020). Therefore, the inhibition of mTORC1 suggests that AZA can switch cells away from protein synthesis and toward autophagy. The ISR is activated in response to diverse stimuli and is characterised by the phosphorylation of eIF2α-S51 by eIF2α kinases (Pakos-Zebrucka et al. 2016). There are four forms of eIF2α kinase: EIF2AK1 (HRI), EIF2AK2 (PKR), EIF2AK3 (PERK), and EIF2AK4 (GCN2). Activation of the ISR results in a decrease in global protein synthesis and the activation of specific genes that work together to restore cellular homeostasis (Pakos-Zebrucka et al. 2016). Phosphorylation of eIF2α-S51 is also a key event in the induction of autophagy (Humeau et al. 2020; Talloczy et al. 2002). Our results demonstrating an increase in eIF2α-S51 phosphorylation suggest that 6-TG and AZA can stimulate eIF2α kinases to reduce protein synthesis and induce autophagy. This aligns with our previous findings, demonstrating that PERK is important in regulating AZA-induced autophagy (Hooper et al. 2019b). Typically, PERK is activated by ER stress (Pakos-Zebrucka et al. 2016); however, in the present study, thiopurines did not induce an ER stress response strong enough to activate *XBP1* splicing. It is, however, possible that thiopurines caused an accumulation of unfolded proteins in the ER and that other eIF2α kinases, in addition to PERK, regulate thiopurine-induced autophagy. Interestingly, 6-MP had only a minor effect on mTORC1 activity or eIF2α-S51 phosphorylation, indicating additional mechanisms of autophagy induction that have yet to be elucidated. This may be relevant considering studies showing that 6-MP is better tolerated than AZA or 6-TG in some patients (Dubinsky 2004).

Our results suggest that thiopurines activate autophagy, in part through the modulation of the mTORC1 and eIF2α signalling pathways, which may work synergistically to inhibit global protein synthesis while increasing autophagic activity; however, they also reveal differences in their mechanisms of action. Secondary events resulting from the metabolism of AZA, such as increased reactive oxygen species (ROS) production, and release of the imidazole derivative may contribute to autophagy induction. In support of this, the depletion of GSH during AZA treatment has been linked to higher ROS (Al Maruf et al. 2014), and higher ROS has been linked to the induction of autophagy, with ROS scavengers leading to a reduction in apoptosis and LC3-II processing (Chaabane and Appell 2016). Mechanistically, ROS can inhibit PI3K signalling (Wen et al. 2019), and it is well-documented that autophagy is inhibited by the PI3K/AKT/mTORC1 pathway (Laplante and Sabatini 2012). AZA is a prodrug of 6-MP (Fig. 1A), synthesised to produce a derivative of 6-MP that has an imidazole ring, extending the drug’s half-life. As much as 12% of AZA can be metabolised to hypoxanthine and imidazole (Fotoohi et al. 2010), and imidazole conjugates have been shown to have PI3K inhibitory activity (Mohan et al. 2016). In contrast, imidazole has been shown to inhibit autophagy in some contexts by impairing the maturation of autophagosomes to lysosomes (Liu et al. 2015); the authors proposed this is due to imidazole being a weak base, which can enter acidic organelles and cause dysfunction by elevated intra-lysosomal pH.

It is also important to consider the direct conversion of 6-TG to its active substrate, 6-TGN, compared with 6-MP and AZA, which undergo a series of enzymatic steps to produce 6-TGN (Fig. 1A). Higher concentrations of 6-TGN are generated from 6-TG treatment compared with AZA and 6-MP (Petit et al. 2008), and 6-TG treated cells exhibit a higher accumulation of metabolites 6-Thio-GMP, GDP, and GTP compared with 6-MP treated cells (Shi et al. 1998). Therefore, 6-TG may have different effects on cellular processes compared with AZA and 6-MP due to the production of higher concentrations of metabolites.

Autophagy is a cell type-specific process; therefore, future work must investigate how thiopurines modulate autophagy in a range of cell types, including those harbouring polymorphisms in genes involved in thiopurine metabolism (Broekman et al. 2014; Roberts et al. 2018) and those harbouring polymorphisms in autophagy genes such as IBD-associated *ATG16L1* and *IRGM* (Barrett et al. 2008). It is critical to explore the effect of thiopurines on autophagy at clinically relevant concentrations in vivo, and further work is required to understand the complex signalling pathways regulated by thiopurines and how they converge on the autophagy molecular machinery. A better understanding of how thiopurines work may lead to improved treatment efficacy and personalised therapies with fewer side effects.

In conclusion, this research demonstrated that thiopurines are strong activators of autophagy, and autophagy induction should be considered among the mechanisms responsible for patient response to thiopurines.

## Supplementary Information

Below is the link to the electronic supplementary material.

Supplementary Fig. 1 mTORC1 immunoblotting replicates (PNG 2.05 MB)

High Resolution Image (TIFF 33973 KB)

Supplementary Fig. 2 eIF2α immunoblotting replicates (PNG 2.17 MB)

High Resolution Image (TIFF 33973 KB)

Supplementary Table 1 List of antibodies (PNG 2.40 MB)

High Resolution Image (TIFF 33973 KB)

## Data Availability

No datasets were generated or analysed during the current study.
