# Supplementary figures and images for: Characterisation of autophagy induction by the thiopurine drugs azathioprine, mercaptopurine and thioguanine in THP-1 macrophages
Source: Naunyn Schmiedebergs Arch Pharmacol. 2024 Nov 1;398(4):4467–78. doi: 10.1007/s00210-024-03563-0 (PMC11978722; doi:10.1007/s00210-024-03563-0)

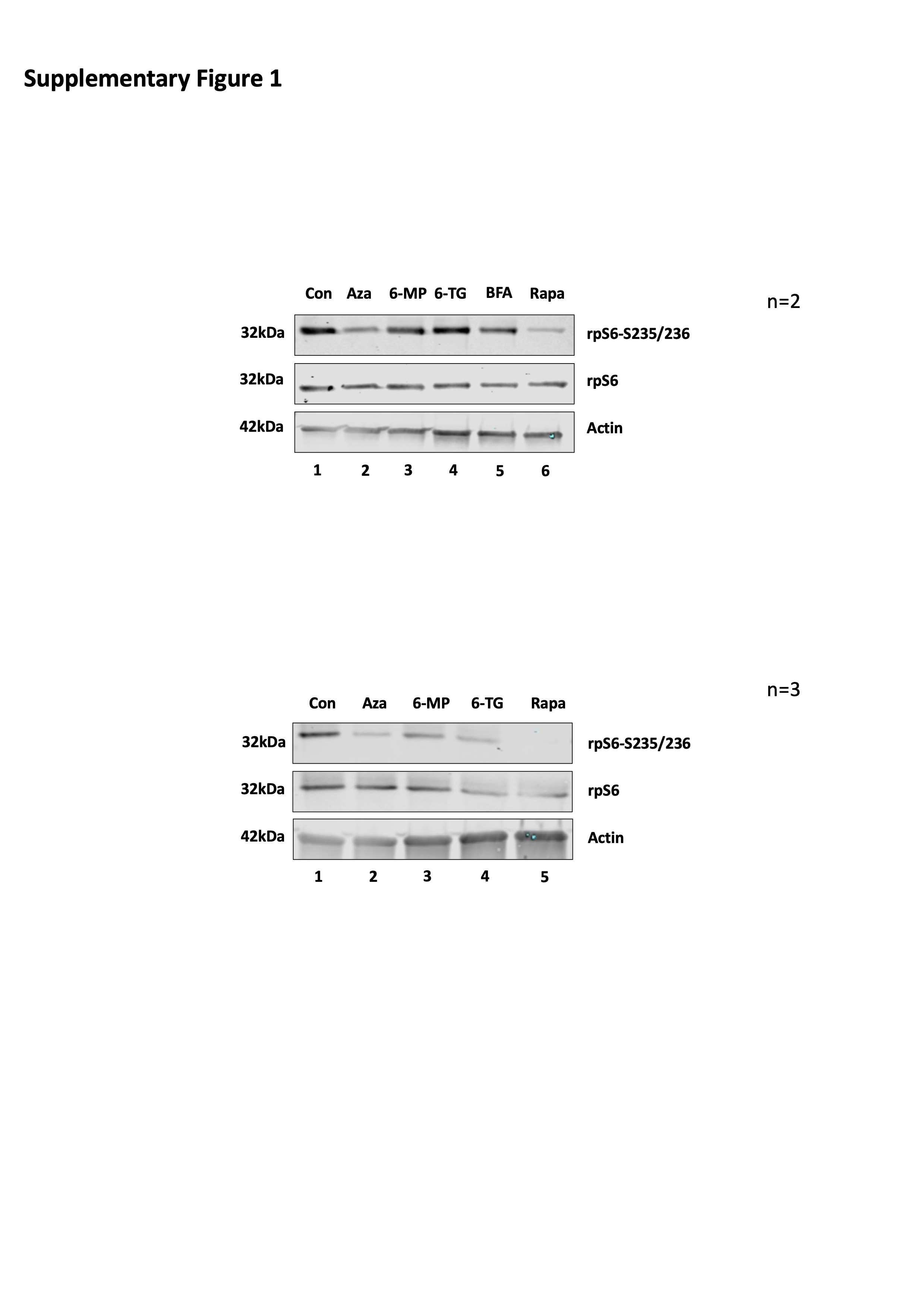

Supplement: Supplementary file 2 — High Resolution Image (TIFF 33973 KB) [file 210_2024_3563_MOESM1_ESM.tiff]

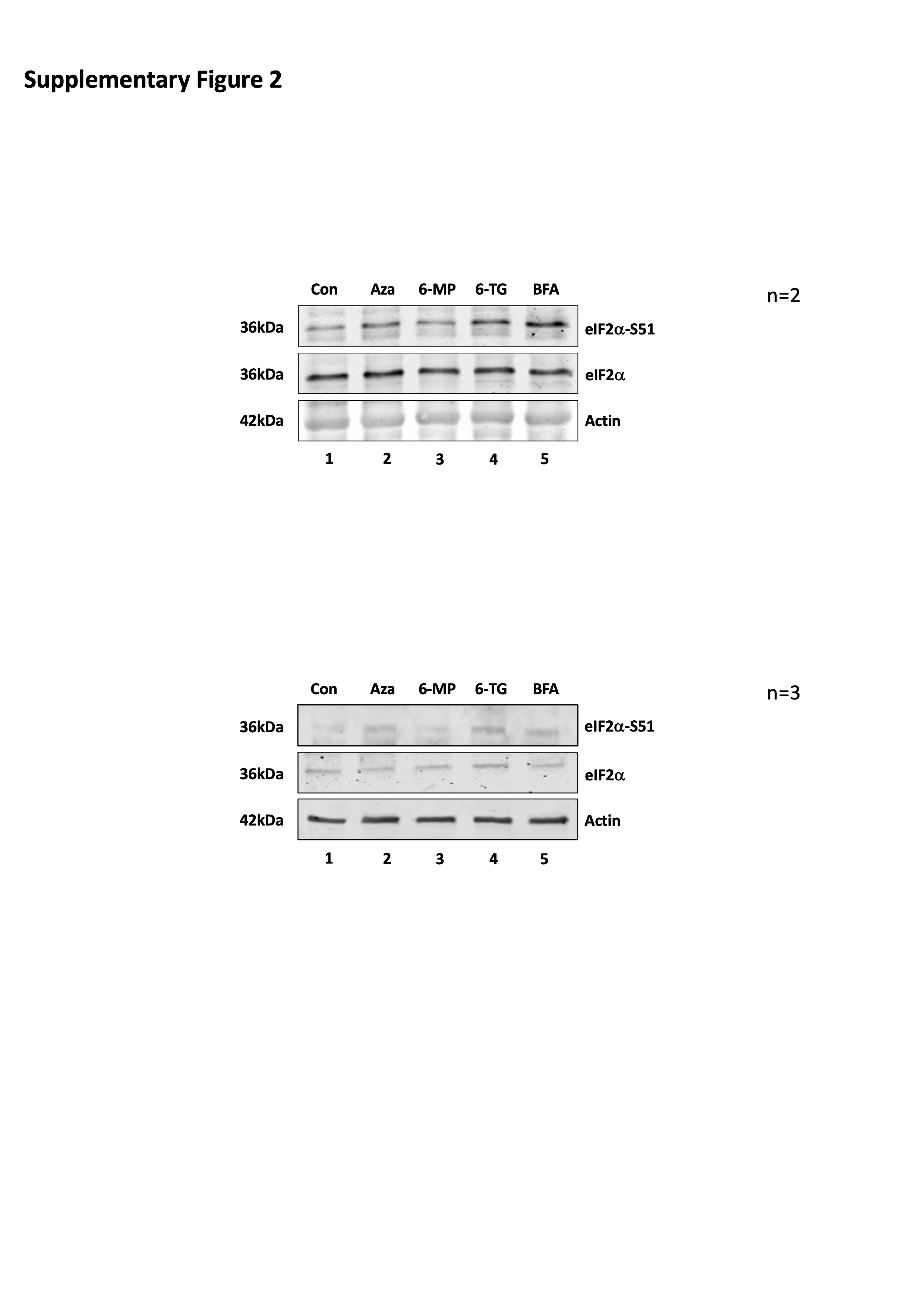

Supplement: Supplementary file 4 — High Resolution Image (TIFF 33973 KB) [file 210_2024_3563_MOESM2_ESM.tiff]

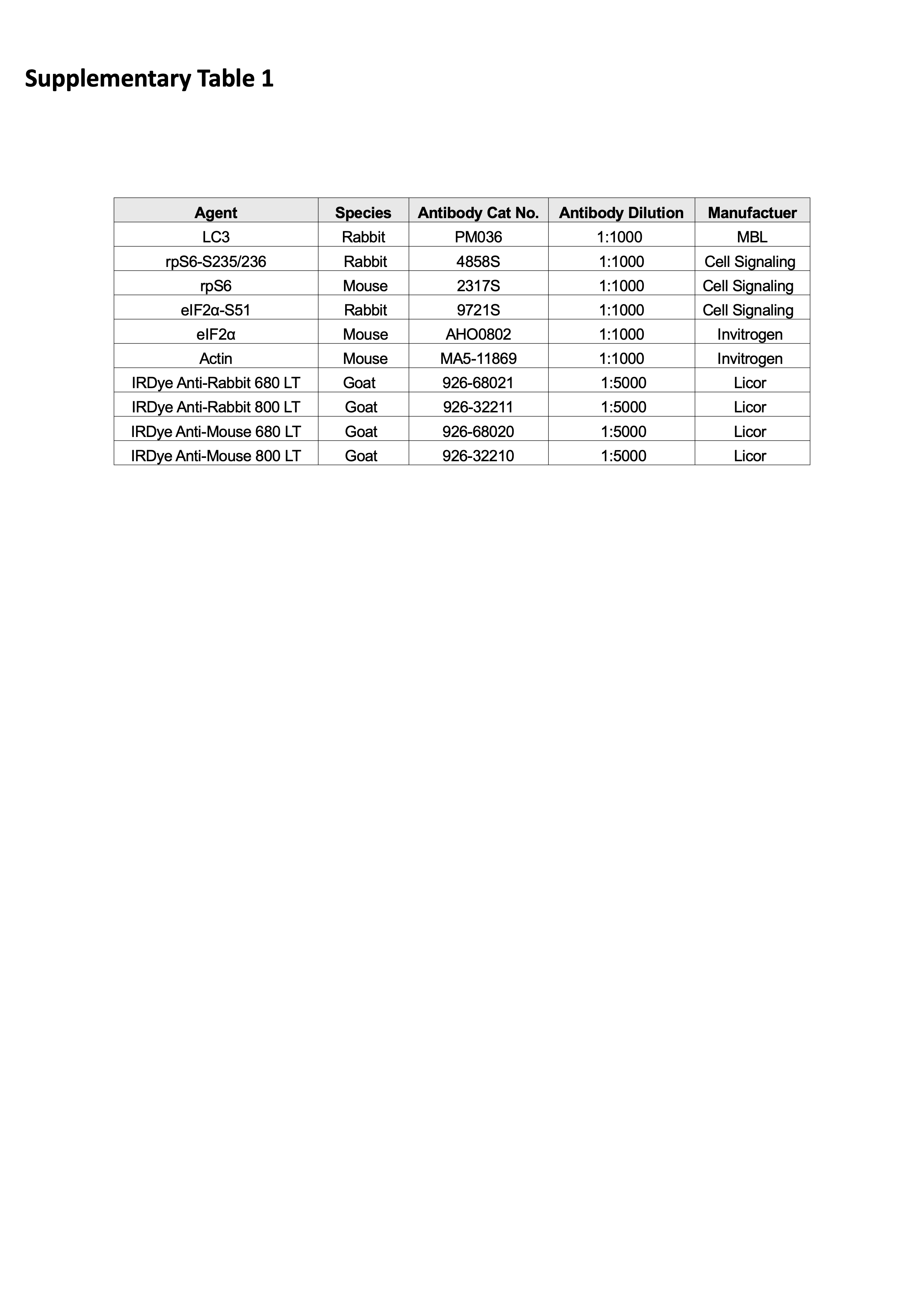

Supplement: Supplementary file 6 — High Resolution Image (TIFF 33973 KB) [file 210_2024_3563_MOESM3_ESM.tiff]
